# Regional citrate anticoagulation “non-shock” protocol with pre-calculated flow settings for patients with at least 6 L/hour liver citrate clearance

**DOI:** 10.1186/s12882-021-02443-6

**Published:** 2021-07-02

**Authors:** Lenar Yessayan, Ryann Sohaney, Vidhit Puri, Benjamin Wagner, Amy Riddle, Sharon Dickinson, Lena Napolitano, Michael Heung, David Humes, Balazs Szamosfalvi

**Affiliations:** 1 Division of Nephrology, Department of Medicine, University of Michigan, 3914 Taubman Center, 1500 E. Medical Center Dr. 5364, Ann Arbor, MI 48109-5364 USA; 2 Division of Acute Care Surgery, Department of Surgery, University of Michigan, Ann Arbor, MI USA

**Keywords:** Veno-venous hemodiafiltration, CKRT, Citrate anticoagulation, Hypocalcemia, Personalized calcium dosing

## Abstract

**Background:**

Regional citrate anticoagulation (RCA) for the prevention of clotting of the extracorporeal blood circuit during continuous kidney replacement therapy (CKRT) has been employed in limited fashion because of the complexity and complications associated with certain protocols. Hypertonic citrate infusion to achieve circuit anticoagulation results in variable systemic citrate- and sodium load and increases the risk of citrate accumulation and hypernatremia. The practice of “single starting calcium infusion rate for all patients” puts patients at risk for clinically significant hypocalcemia if filter effluent calcium losses exceed replacement. A fixed citrate to blood flow ratio, personalized effluent and pre-calculated calcium infusion dosing based on tables derived through kinetic analysis enable providers to use continuous veno-venous hemo-diafiltration (CVVHDF)-RCA in patients with liver citrate clearance of at least 6 L/h.

**Methods:**

This was a single-center prospective observational study conducted in intensive care unit patients triaged to be treated with the novel pre-calculated CVVHDF-RCA “Non-shock” protocol. RCA efficacy outcomes were time to first hemofilter loss and circuit ionized calcium (iCa) levels. Safety outcomes were surrogate of citrate accumulation (TCa/iCa ratio) and the incidence of acid-base and electrolyte complications.

**Results:**

Of 53 patients included in the study, 31 (59%) had acute kidney injury and 12 (22.6%) had the diagnosis of cirrhosis at the start of CVVHDF-RCA. The median first hemofilter life censored for causes other than clotting exceeded 70 h. The cumulative incidence of hypernatremia (Na > 148 mM), metabolic alkalosis (HCO3- > 30 mM), hypocalcemia (iCa < 0.9 mM) and hypercalcemia (iCa > 1.5 mM) were 1/47 (1%), 0/50 (0%), 1/53 (2%), 1/53 (2%) respectively and were not clinically significant. The median (25th–75th percentile) of the highest TCa/iCa ratio for every 24-h interval on CKRT was 1.99 (1.91–2.13).

**Conclusions:**

The fixed citrate to blood flow ratio, as opposed to a titration approach, achieves adequate circuit iCa (< 0.4 mm/L) for any hematocrit level and plasma flow. The personalized dosing approach for calcium supplementation based on pre-calculated effluent calcium losses as opposed to the practice of “one starting dose for all” reduces the risk of clinically significant hypocalcemia. The fixed flow settings achieve clinically desirable steady state systemic electrolyte levels.

## Introduction

The 2012 Kidney Disease Improving Global Outcomes (KDIGO) acute kidney injury (AKI) guideline advocated for citrate as first-line anticoagulation method for continuous kidney replacement therapy (CKRT) [1]. Regional citrate anticoagulation (RCA) has been shown to prolong circuit life over heparin [2] and this was recently confirmed in a large multicenter randomized controlled trial [3]. However, unfractionated heparin remains widely used for CKRT circuit anticoagulation [4, 5]. The main disadvantage of heparin is that it causes systemic anticoagulation in addition to circuit anticoagulation and increases the risk of hemorrhagic complications [6–8]. Potential reasons for the slow adoption of RCA include the perceived complexity of its use, the need for rigorous blood monitoring and for frequent RCA or calcium infusion adjustments, and the variability in published approaches [9].

Since January 2018 we have implemented a novel CKRT-RCA program for intensive care unit (ICU) providers at our institution based on post-dilution continuous veno-venous hemodiafiltration (CVVHDF) that allows us to treat all patients with RCA without contraindications. Patients are first triaged to one of two novel protocols with unique pre-calculated flow settings and CKRT fluid compositions, based on whether citrate metabolism is presumed to be sufficiently present (referred to as “non-shock” protocol; systemic citrate clearance ≥6 L/h) or absent (referred to as “shock” protocol; systemic citrate clearance < 6 L/h) [10]. The triage is guided by clinical assessment for the presence of severe liver failure and/or shock and by laboratory criteria including markedly elevated lactate or INR. Once the patient is triaged to the “non-shock” protocol presented here, all CKRT-RCA flow settings are obtained from pre-calculated tables incorporating a high, fixed citrate to blood flow ratio and a personalized initial calcium infusion rate which simplifies RCA management and diminishes the risk of hypocalcemia.

Here we report on the design, efficacy, and safety of the CVVHDF-RCA “non-shock” protocol. The approach is based on principles of CKRT-RCA protocols designed for near automated delivery we have described before, specifically optimized for use on the Prismaflex, PrisMax (Baxter) or the Multifiltrate Pro (Fresenius) CKRT machines with equal use of separate dialysate- and replacement fluid scales, for patients with liver clearance of citrate ≥6 L/h (about 80–90% of patients will be in this group in most ICU CKRT populations).

## Material and methods

### Study design and participants

We conducted a prospective observational study of patients triaged to the CVVHDF-RCA “non-shock” protocol in 5 Adult Intensive Care Units at the University of Michigan between March and September 2018. The study was approved by the Institutional Review Board of University of Michigan (IRB # HUM00029545) and informed consent was waived for reasons that include the observational nature of the study and the feasibility constraint of obtaining consent from all patients triaged to the protocol.

### CKRT procedure

The Prismaflex CKRT machine is used in post-dilution CVVHDF mode. Acid citrate dextrose anticoagulant flow (QACDA) is delivered by the pre-blood pump (PBP) and bicarbonate-buffered dialysate- (QD) and post-dilution replacement fluids (QRF) are delivered by their respective pumps at an equal rate to ensure that the fluid bags on the separate scales empty at the same time decreasing the frequency of bag-change interventions. The calcium chloride solution (136 mmol/L CaCl_2_ in 0.9% saline) is infused at the end of the return limb of the blood circuit by a separate infusion pump. HF1400 hemofilter sets were used for all treatments in the “non-shock” protocol.

CKRT prescribing is designed to support ICU providers without a special expertise in solute kinetic analysis and consists of mechanistically following several steps. First, patients are triaged to the citrate metabolism presumed present pathway (> 6 L/h) (which is sufficient to keep the systemic citrate level ≤ 2.5 mM using the “non-shock” protocol) if the following criteria are satisfied: systemic lactate < 10 mM in patients with shock, no requirement of dextrose drips to prevent hypoglycemia or fresh frozen plasma (FFP) to keep the INR < 3 in patients with cirrhosis, and no diagnosis of acute liver failure/shock liver during the current admission close to the time of CKRT-RCA start. Second, flows are selected: blood flow (QB), QACDA, QD, QRF from Table 1 based on body weight (10 kg increments) for a prescribed effluent dose about 30 ml/kg/hr. Third, the initial calcium chloride solution (136 mmol/L CaCl_2_ in 0.9% saline) flow rate (QCa) is selected from a table based on the patient’s systemic albumin level and the total effluent flow rate (Table 2).

**Table 1:** CVVHDF RCA: FIXED CKRT Settings Selected Based on the Patient’s Current Weight

Weight	Effluent Flow, QEFF ml/hour	Blood Flow, QB ml/min	Citrate Flow, QACDA ml/hour	Dialysate Flow, QD ml/hour	Post-Dilution Flow, QRF ml/hour
**<=50 kg**	**1250+**	**100**	**250**	**500**	**500**
**51–60 kg**	**1550+**	**100**	**250**	**650**	**650**
**61–70 kg**	**1850+**	**100**	**250**	**800**	**800**
**71–80 kg**	**2150+**	**100**	**250**	**950**	**950**
**81–90 kg**	**2400+**	**150**	**300**	**1050**	**1050**
**91–100 kg**	**2700+**	**150**	**300**	**1200**	**1200**
**101–110 kg**	**3000+**	**150**	**300**	**1350**	**1350**
**111–120 kg**	**3300+**	**150**	**300**	**1500**	**1500**
**121–130 kg**	**3600+**	**150**	**300**	**1650**	**1650**
**131–140 kg**	**3900+**	**150**	**300**	**1800**	**1800**
**> = 141 kg**	**4200+**	**150**	**300**	**1950**	**1950**

*Abbreviations*: *QB* Blood flow rate, *QACDA* Acid citrate dextrose anticoagulant flow rate, *QD* Bicarbonate-buffered dialysate flow rate, *QRF* Post-dilution replacement fluid flow rate, *QEFF* Filter effluent flow rate, *kg* kilogram, *ml* milliliter, *min* minute

**Table 2:** Initial 136 mM CaCl_2_-infusion (ml/h) for Goal Systemic iCa 1.15 mM for “Non-Shock” Protocol

	Systemic Albumin Level (g/dL)									
Effluent Flow ml/hour	0.0–0.7 g/dL	0.8–1.2 g/dL	1.3–1.7 g/dL	1.8–2.2 g/dL	2.3–2.7 g/dL	2.8–3.2 g/dL	3.3–3.7 g/dL	3.8–4.2 g/dL	4.3–4.7 g/dL	4.8–5.2 g/dL
**<=1400**	16	17	17	18	19	19	20	20	21	21
**1401–1700**	20	20	21	22	23	23	24	25	25	26
**1701–2000**	23	24	25	26	27	28	28	29	30	31
**2001–2300**	26	27	28	30	31	32	32	33	34	35
**2301–2600**	32	33	34	35	37	38	39	40	41	42
**2601–2900**	35	37	38	39	41	42	43	44	45	46
**2901–3200**	38	40	42	43	44	46	47	48	50	51
**3201–3500**	42	43	45	47	48	50	51	53	54	55
**3501–3800**	45	47	48	50	52	54	55	57	58	60
**3801–4100**	48	50	52	54	56	57	59	61	63	64
**4101–4400**	51	53	55	57	59	61	63	65	67	68

Footnote: For patients with expected good to fair citrate metabolism the initial Ca infusion rate is prescribed by the nephrology team based on the effluent flow row (=QACDA+ QD+ QRF+ Net UF) and the last measured systemic serum albumin column. Calcium replacement is the product of the plasma Ca-clearance times the goal serum total Ca level. The patient’s serum albumin level determines the target plasma total Ca level needed to keep the plasma iCa around 1.05–1.25 mmol/L with systemic citrate clearance estimated at about 15 L/hour and systemic citrate level estimated in the 0.7–1.4 mM range. To target a higher systemic iCa of 1.3 mM (at the ICU team’s discretion) simply multiply by 1.13 the initial Ca-infusion rate derived from Table 2 (which is designed for goal systemic iCa 1.15 mM)

*Abbreviations*: *g/dL* grams per deciliter, *ml/hour* milliliter per hour, *QCa* Calcium infusion rate, *QACDA* Acid citrate dextrose anticoagulant infusion rate, *QD* dialysate flow rate, *QRF* replacement fluid flow rate, *Net UF* machine set net ultrafiltration rate

### CKRT related laboratory measurements

Systemic iCa was drawn via arterial or venous access line every 2 h during the first 6 h of CKRT and every 6 h thereafter. Circuit iCa was drawn post-filter and before the post-dilution infusion from the return blood sample port every 12 h. Other laboratory measurements included daily serum sodium, potassium, chloride, carbon dioxide, blood urea nitrogen, creatinine, glucose, calcium phosphorus, magnesium, and albumin. During the first 6 h of CVVHDF-RCA the systemic iCa is monitored every 2 h to detect ionized hypocalcemia due to citrate accumulation if a patient with systemic citrate clearance < 6 L/h was incorrectly triaged to the “Non-shock” protocol.

Finally, during CVVHDF-RCA therapy the QCa is adjusted in increments of +/− 10–20% of the current QCa based on how the systemic iCa measured every 6 h compares to the goal systemic iCa (either 1.05–1.25 mM, see Table 3 or 1.2–1.4 mM, see Table 4).

**Table 3:** 136 mM CaCl_2_ Infusion Rate Change Based on Systemic iCa Every 6 h: GOAL Systemic iCa 1.15 mmol/L

	The patient’s ionized calcium level checked every 6 h				
	Less than < 0.95 mmol/L	0.95–1.04 mmol/L	1.05–1.25 mmol/L	1.26–1.4 mmol/L	More than > 1.4 mmol/L
Current Ca-infusion Flow Rate mL/h	Increase Rate + 20%; notify ICU and Nephro fellows	Increase Rate + 10%	No Change	Reduce Rate −10%	Reduce Rate −20%; notify ICU and Nephro fellows
**<=15**	+ 2 ml/h	+ 1 ml/h	No change	−1 ml/h	−2 ml/h
**16–25**	+ 4 ml/h	+ 2 ml/h	No change	− 2 ml/h	−4 ml/h
**26–35**	+ 6 ml/h	+ 3 ml/h	No change	−3 ml/h	−6 ml/h
**36–45**	+ 8 ml/h	+ 4 ml/h	No change	−4 ml/h	−8 ml/h
**46–55**	+ 10 ml/h	+ 5 ml/h	No change	− 5 ml/h	− 10 ml/h
**56–65**	+ 12 ml/h	+ 6 ml/h	No change	−6 ml/h	− 12 ml/h
**66–75**	+ 14 ml/h	+ 7 ml/h	No change	−7 ml/h	− 14 ml/h
**76–85**	+ 16 ml/h	+ 8 ml/h	No change	−8 ml/h	− 16 ml/h
**86–95**	+ 18 ml/h	+ 9 ml/h	No change	−9 ml/h	−18 ml/h
**96–105**	+ 20 ml/h	+ 10 ml/h	No change	− 10 ml/h	− 20 ml/h

*Abbreviations*: *mL/h* milliliter per hour, *mmol/L* millimole per liter

**Table 4:** 136 mM CaCl_2_ Infusion Rate Change Based on Systemic iCa Every 6 h: GOAL Systemic iCa 1.3 mmol/L

	The patient’s ionized calcium level checked every 6 h				
	Less than < 1.1 mmol/L	1.1–1.19 mmol/L	1.2–1.4 mmol/L	1.41–1.55 mmol/L	More than > 1.55 mmol/L
Current Ca-infusion Flow Rate mL/h	Increase Rate + 20%; notify ICU and Nephro fellows	Increase Rate + 10%	No Change	Reduce Rate −10%	Reduce Rate − 20%; notify ICU and Nephro fellows
**<=15**	+ 2 ml/h	+ 1 ml/h	No change	−1 ml/h	−2 ml/h
**16–25**	+ 4 ml/h	+ 2 ml/h	No change	− 2 ml/h	− 4 ml/h
**26–35**	+ 6 ml/h	+ 3 ml/h	No change	−3 ml/h	− 6 ml/h
**36–45**	+ 8 ml/h	+ 4 ml/h	No change	− 4 ml/h	−8 ml/h
**46–55**	+ 10 ml/h	+ 5 ml/h	No change	− 5 ml/h	− 10 ml/h
**56–65**	+ 12 ml/h	+ 6 ml/h	No change	− 6 ml/h	− 12 ml/h
**66–75**	+ 14 ml/h	+ 7 ml/h	No change	− 7 ml/h	− 14 ml/h
**76–85**	+ 16 ml/h	+ 8 ml/h	No change	−8 ml/h	− 16 ml/h
**86–95**	+ 18 ml/h	+ 9 ml/h	No change	− 9 ml/h	−18 ml/h
**96–105**	+ 20 ml/h	+ 10 ml/h	No change	− 10 ml/h	− 20 ml/h

*Abbreviations*: *mL/h* milliliter per hour, *mmol/L* millimole per liter

### Solutions used with the post-dilution CVVHDF-RCA “non-shock” protocol

In the USA there are no FDA approved citrate solutions for CKRT-RCA. To avoid the cost and uncertain availability of compounded citrate solutions, we use USP Acid Citrate Dextrose Anticoagulant (ACDA). ACDA is FDA-approved for anticoagulation during plasmapheresis and has a published record of clinical use in CKRT-RCA off label. Of note, a 0.5% sodium citrate containing pre-dilution replacement fluid (Regiocit) was recently authorized by the FDA for emergency use for no longer than the duration of the COVID-19 public health emergency.

The default CKRT fluid (dialysate and replacement) sodium level is 136 mmol/L, bicarbonate level is 25 mmol/L, potassium is 4 mmol/L and phosphate is 1.36 mmol/L; these can be adjusted as needed (Table 5) in institutions with an established program for pharmacy to spike CKRT fluid bags. For institutions which do not have pharmacy support for spiking, recommended fluid compositions are also shown in Table 5.

**Table 5:** Commercially available citrate solution and CKRT fluids and compounded Ca-infusion used with the “non-shock” CVVHDF-RCA protocol

Solute	ACDA Citrate 113 mM	CKRT Fluid (1) BBraun 4553 2 K/25Bic	CKRT Fluid (2) BBraun 4556 4 K/25Bic	136 mM CaCl_**2**_ in 0.9% Saline
**Calcium (mM)**		0	0	136
**Magnesium (mM)**		0.75	0.75	0
**Chloride (mM)**		114.5	116.5	395
**Glucose (mM)**	124	0	0	0
**Sodium (mM)**	225	136	136	123
**Citric Acid (mM)**	38			
**Citrate**^**3−**^ **(mM)**	75			
**Potassium (mM)**		2	4	
**Bicarbonate (mM)**		25	25	

Footnote: In our institution, we customize the commercial CKRT fluid 1 by spiking with K-phosphate or K-chloride to final K 2, 3, or 4 mmol/L and phosphate 2.1 or 4.2 mg/dL and with NaHCO3 (default spiking: none) to final Na 136 (no added HCO3), 141 or 146 mM and HCO3 25, 30, or 35 mM (for patients who need a systemic HCO3 level > 25 and/or have moderately impaired citrate metabolism with or without moderate lactic acidosis < 10 mM). In hospitals without pharmacy support for CKRT fluid compounding the CKRT fluid 2 can be used for 4 K CKRT and phosphate could be supplemented as 15 mmol of Na-phosphate intravenous piggyback every 8 to 12 h for most patients

*Abbreviations*: *mM* millimolar, *ACDA* Acid citrate dextrose A, *K* Potassium, *Bic* Bicarbonate, *CaCl*_*2*_ Calcium chloride

With the fixed Tables 1 and 2 “non-shock” CVVHDF-RCA settings the 136 mM sodium (Na) CKRT fluids result in around 140 mM systemic Na levels; the 25 mM bicarbonate fluids result in a systemic bicarbonate around 22–25 mM. Glucose-free CKRT fluids and Ca-infusion in combination with the glucose containing ACDA solution result in neutral CKRT glucose mass balance at normal systemic glucose levels; CKRT fluids with 0.75 mM (1.5 mEq/L) magnesium (Mg) level prevent hypomagnesemia. Ca-free CKRT fluids (as opposed to Ca-containing CKRT fluids) avoid reversing the RCA effect on the venous end of the fiber bundle and hence contribute to stable Ca-clearance on the filter during days of use with fixed settings. The compounded calcium chloride solution was produced in our local hospital pharmacy by adding 12.5 × 10-ml amps of 10% CaCl_2_ to 0.5 L of 0.9% physiologic saline.

### Systemic citrate level simulations and CVVHDF-RCA Ca clearance calculations for Tables 1 and 2

The plasma clearance of citrate- and calcium on the Prismaflex circuit with the HF1400 hemofilter was calculated by adapting post-CVVHDF clearance equations described in the literature and using a Microsoft Excel clearance calculator [11]. A single pool, fixed volume kinetic equation described by Szamosfalvi et al (US Patent Application 2008, 0015487) and validated by Zheng [12] was used to generate the systemic citrate accumulation curves.

$$ {C}_{(t)}={C}_{(0)}\times {e}^{-\left(\frac{K_f+{K}_b}{V}\times t\right)}+\frac{G}{K_f+{K}_b}\times \left(1-{e}^{-\left(\frac{K_f+{K}_b}{V}\times t.\right)}\right) $$

Where *C*_(*t*)_ is the systemic plasma citrate concentration in mmol/L, *K*_*f*_ is dialyzer clearance in L/hr., *K*_*b*_ is body citrate clearance in L/hr., *V* is the volume of distribution of citrate (~ ECF space), *t* is the time on CKRT in hours and *G* is the citrate load in mmol/hr. The simulated curves (Fig. 3) are based on an average non-shock CVVHDF-RCA prescription (QB 150 ml/min, QACDA 300 ml/min, QD 1.2 L/h, QRF 1.2 L/h and estimated effluent flow about 2.8 L/hr), a patient with extracellular fluid volume of 15 L + 7 L edema, systemic Hb 7.5 g/dL and various values of estimated body (liver) clearance of citrate (15 L/h usual with compensated liver cirrhosis, 6 L/h severely impaired but sufficient to avoid citrate toxicity and 0 L/h completely absent).

### Data sources

Demographics, clinical variables, and laboratory data during the first 96 h of CVVHDF-RCA treatment were collected from the electronic medical records. Filter clotting data and reasons for disconnection were collected from data recorded by the ICU nurse in the electronic record. Data was collected by two research fellows and transcribed into Excel files by a research resident.

### Study variables

RCA effectiveness in decreasing clotting was measured in terms of time to first hemofilter loss due to clotting as recorded by the ICU nurses and based on the established surrogate variable, circuit iCa levels. Hemofilter life was defined as the time elapsed between the start of the blood flow through the filter and the time when blood was unable to pass through the filter due to clot formation or obstruction of the filter [13]. Hypocalcemia was defined as systemic iCa < 0.9 mmol/L; clinically significant hypercalcemia as iCa > 1.5 mmol/L; Hypernatremia as plasma Na > 148 mmol/L; metabolic alkalosis as HCO3- > 30 mmol/L and pH > 7.45 and clinically significant hypophosphatemia as *P* < 2.0 mg/dL.

Electrolyte complications after CKRT initiation were *attributable* to CVVHDF-RCA if the following criteria were met for each of the following variable: iCa < 0.9 mmol/L; iCa > 1.5 mmol/L in the absence of exogenous calcium administration beyond dictated by the protocol; serum Na > 148 mmol/L and > 5 mEq/L rise in systemic Na above the prescribed CKRT fluid Na level in the absence of hypertonic intravenous Na infusion; HCO3- > 30 mmol/L with pH > 7.45 in the absence of exogenous bicarbonate administration; P < 2.0 mg/dl.

### Study outcomes

The primary outcome was hemofilter life. Secondary outcomes were surrogate of citrate efficacy (circuit iCa), surrogate of citrate accumulation (tCa/iCa ratio), prevalent electrolyte and acid-base trends, the cumulative incidence of acid-base and electrolyte disturbances.

### Statistical analysis

Statistical analysis was performed using MedCalc Statistical Software version 19.1.5 (MedCalc Software bv, Ostend, Belgium; https://www.medcalc.org; 2020). Categorical data were reported as frequencies ± percentages and continuous data as mean ± standard deviation (SD) or median (interquartile range (IQR)) when non normally distributed. The 96-h clotting/clogging-free hemofilter survival rates were calculated using the Kaplan-Meier product limit estimator. Quantitative data trends for select solute levels are presented in boxplots.

## Results

A total of 56 patients with up to 96-h sessions of CVVHDF-RCA satisfied inclusion criteria. Three patients whose filter life was less than 4 h were excluded leaving 53 patients for final analysis. Demographics and baseline characteristics are shown in Table 6. Twenty-one patients were started on a blood flow of 100 ml/min and 32 on a blood flow of 150 ml/min. Characteristics of the initial prescriptions are shown in Table 7.

**Table 6:** Demographics and Baseline Characteristics

Demographics and Baseline Characteristics	Value
Age mean ± SD	60.0 ± 14.8
Male (n, %)	35 (66.0%)
Cause of admission	
Medical (n, %)	36 (67.9%)
Surgical (n, %)	17 (32.1%)
ESKD (n, %)	22 (41.5%)
CKD (n, %)	34 (64.15%)
Total AKI (n, %)	31 (58.5%)
ANCA vasculitis	1
ATN	24
TLS	2
Cardiorenal	2
HRS	1
Lupus nephritis	1
AKI/CKD (n, %)	16 (30.2%)
AKI with no CKD (n, %)	15 (28.3%)
PVD (n, %)	14 (27.5%)
CHF (n, %)	24 (45.3%)
Hyperlipidemia (n, %)	25 (47.2%)
CAD (n, %)	18 (34.0%)
HTN (n, %)	35 (66.0%)
COPD (n, %)	5 (9.4%)
DM (n, %)	19 (35.8%)
Cirrhosis (n, %)	12 (22.6%)
Cancer (n, %)	10 (18.9%)

*Abbreviations*: *ESKD* End stage kidney disease, *CKD* Choric kidney disease, *AKI* Acute kidney injury, *PVD* Peripheral vascular disease, *CHF* Congestive heart failure, *CAD* Coronary artery disease, *HTN* Hypertension, *COPD* Chronic obstructive pulmonary disease, *DM* Diabetes

**Table 7:** Initial Prescription Settings

Blood Flow Rate (ml/min)	Number of patients	Calcium flow rate (ml/h)	Citrate flow rate (ml/h)	Dialysate fluid flow rate (ml/h)	Replacement fluid flow rate (ml/h)
100	21	33.7 ± 1.8	250.0 ± 1.5	952.4 ± 59.7	952.4 ± 59.7
150	32	46.0 ± 1.5	298.4 ± 1.2	1334.4 ± 48.4	1334.4 ± 48.4

**Fig. 1:**
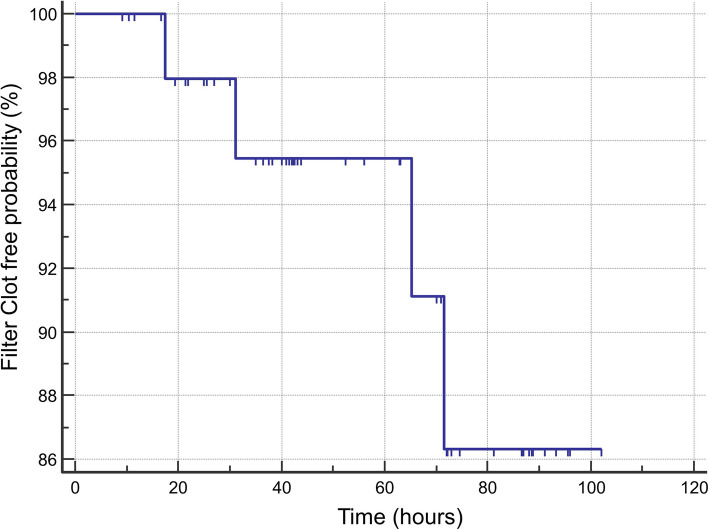
Kaplan-Meier survival curve for hemofilter life

The median first CKRT circuit duration was 43.8 h (IQR, 30.8–82.5 h). The median hemofilter life exceeded 72 h (86% of the hemofilters were clot free at 72 h). The first hemofilter clotting free probability for 53 patients censored for other causes of interruption is shown in Fig. 1. The mean circuit iCa was 0.36 ± 0.05 mmol/L. Causes of circuit interruption included hemofilter clotting 4/53 (7.5%), catheter dysfunction (4/53, 7.5%), death or withdrawal of care (2/53, 3.8%), loss of dialysis access (1/53,1.8%), need for procedures (19/53, 35.9%), or discontinued (18/53, 33.8%) for other reasons that included recovery of kidney function, transition to hemodialysis, machine malfunction or per physician order.

**Fig. 2:**
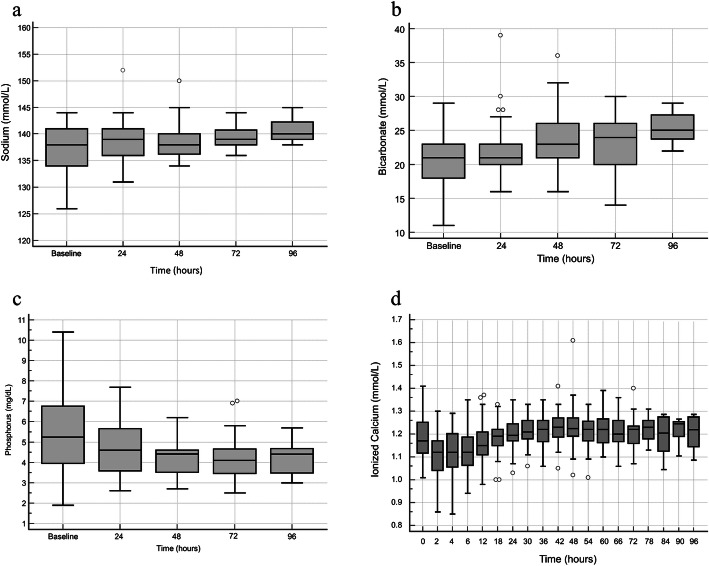
Standard boxplots of systemic plasma sodium-, bicarbonate-, phosphorus-, and ionized calcium concentration during CVVHDF-RCA

Standard boxplots for systemic sodium, HCO3, phosphorus, and iCa levels are shown in Fig. 2. The cumulative incidence of hypernatremia attributable to CVVHDF-RCA was 0/47 (0%). One patient developed hypernatremia; however, this was not attributable to CVVHDF-RCA. The patient was receiving hypertonic saline infusion (sodium bicarbonate 8.4%, 1000 mEq/L) at a rate of 50 ml/hr. for 4 h prior to CKRT initiation which was continued for the duration of ICU therapy to manage chronic respiratory acidosis. The cumulative incidence of metabolic alkalosis and hypophosphatemia *attributable* to CVVHDF-RCA were 0/50 (0%) and 0/52 (0%) respectively. The cumulative incidence of hypocalcemia was 1/53 (2%). The low systemic iCa values were 0.85, 0.86 mmol/L and occurred at hours 2 and 4 from CKRT initiation. In this patient the pre-calculated calcium dosing recommendation from Table 2 was inadvertently not followed. The systemic iCa started to normalize at 6 h after following the calcium dosing recommendations. The cumulative incidence of hypercalcemia was 1/53 (2%). A systemic iCa of 1.6 mmol/L was recorded in one patient at 48 h from CKRT initiation. Since no changes were made in CKRT prescription, this was suspected to be lab error and repeat iCa sent immediately after (24 min) was within normal range (1.24 mmol/L). Table 8 shows measures of central tendency and spread of the highest tCa/iCa ratio recorded in any 24-h period. The maximum recorded tCa/iCa ratio in any 24-h period was 2.55 suggesting systemic citrate accumulation was maintained within a clinically acceptable level.

**Table 8:** Measures of central tendency and spread of the highest tCa/iCa ratio recorded in any 24-h period while on “Non-shock” protocol CVVHDF-RCA

Interval	N	Median	25th–75th percentile	Minimum	Maximum
**First 24 h**	50	2.00	1.92 to 2.16	1.69	2.55
**25 to 48 h**	38	1.98	1.90 to 2.10	1.72	2.46
**49 to 72 h**	22	2.00	1.90 to 2.08	1.76	2.50
**73 to 96 h**	10	2.04	1.92 to 2.13	1.90	2.21

**Fig. 3:**
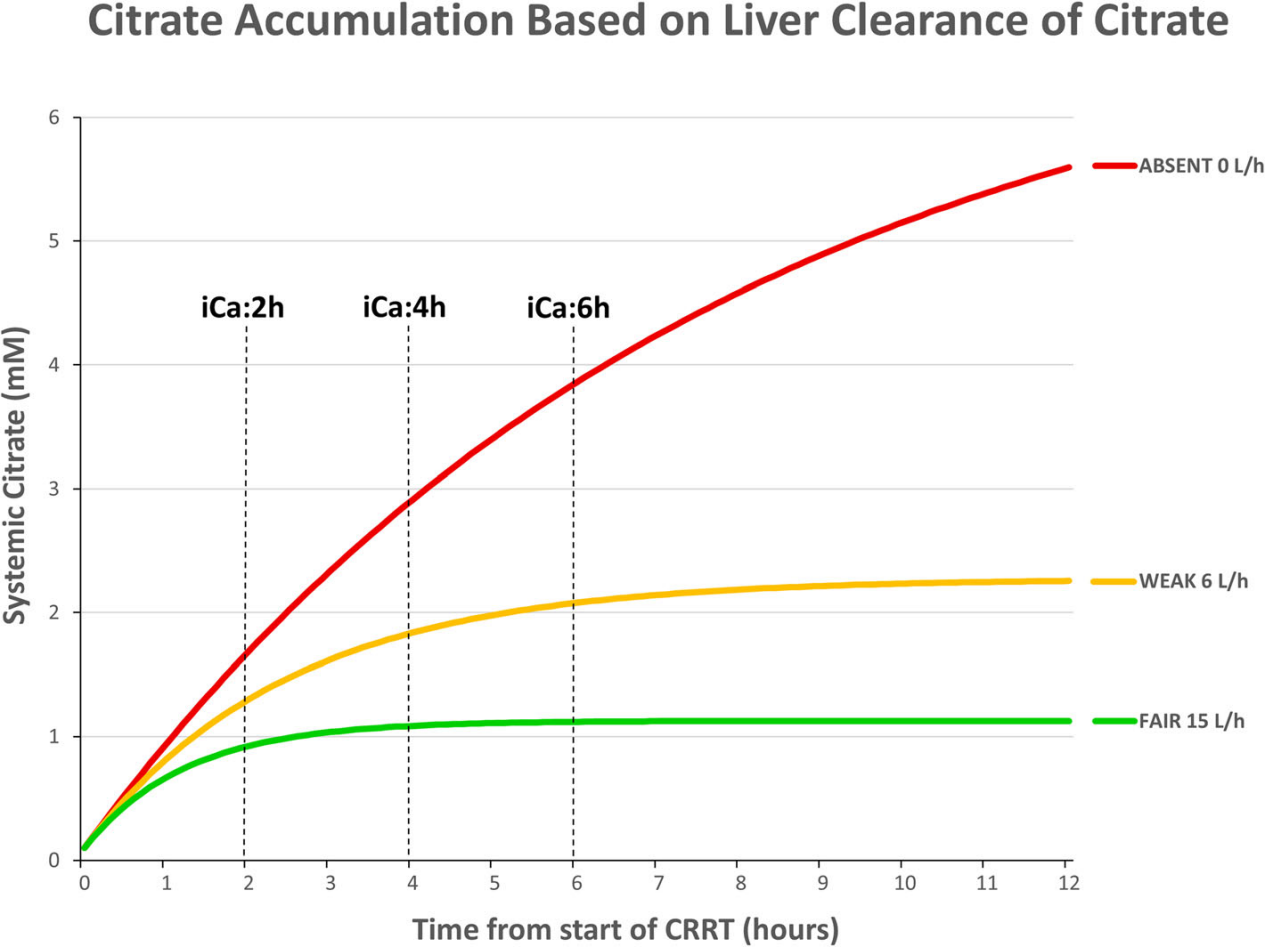
Predicted systemic citrate level kinetic curves using ACDA 300 ml/h, QB150 ml/min, QD 1200/QRF 1200 ml/h and net UF 200 ml/h settings with 15 L/h (green), 6 L/h (yellow) and 0 L/h (red) liver clearance of citrate

The results of simulations of systemic citrate levels at three levels of body citrate clearance (15 L/h, 6 L/h, and 0 L/hr) and CVVHDF-RCA fixed flow settings of QB150 ml/min, QACDA 300 ml/h, QD/QRF 1.2/1.2 L/h are shown in Fig. 3. Systemic citrate levels remain < 2.5 mM within the first 6 h of CKRT start when body citrate clearance is at least 6 L/hr. When body clearance of citrate is set to 0 L/h, systemic citrate may exceed 2.5 mM as early as 4 h from CKRT initiation with the “Non-shock” setting prescriptions. The curves show that by 6 h after CKRT start, a patient either reaches steady state with manageable citrate levels ≤2.5 mM or will develop citrate accumulation (detected by a total Ca (tCa; mM) to iCa (mM) ratio ≥ 2.5 or by a drop in systemic iCa of more than 0.3 mmol/L) to warrant a prescription change to the “shock” protocol settings.

## Discussion

This study describes the delivery of a simplified, novel post-dilution CVVHDF-RCA “non-shock” protocol that utilizes a fixed citrate to blood flow ratio with personalized calcium infusion dosing based on tables derived through kinetic analysis. This approach enables ICU providers without special expertise in solute kinetic analysis to use CKRT-RCA with confidence in critically ill patients with underlying conditions that include sepsis and/or cirrhosis but with maintained minimal liver citrate clearance of at least 6 L/h. The median hemofilter life exceeded 70 h in this study. The reported cases of electrolyte abnormalities were rare and not clinically significant. There are many unique aspects of this approach, all introduced to make CKRT-RCA simpler and safer for ICU providers:

1. Simple pre-calculated initial calcium infusion dosing selected according filter effluent flow rate and systemic albumin level that allows for net neutral calcium balance on the CKRT circuit and completely abrogates the risk of clinically significant hypocalcemia secondary to calcium losses on the filter.
2. Fixed citrate-to-blood flow ratio that decreases variability in systemic iCa secondary to frequent citrate titration and variable systemic citrate load.
3. The high citrate-to-blood flow ratio ensures that adequate circuit anticoagulation is achieved even pre-filter, regardless of systemic hemoglobin level and hence plasma flow at a fixed blood flow. This results in circuit iCa < 0.4 mM and no adjustment to the ACDA rate resulting in reduced ICU nurse workload especially if circuit iCa checks are abandoned as unnecessary.
4. Effluent flow which is about 20–50% of the blood flow that clears 30–60% of the free [citrate]^3−^ and [citrate-Ca]^−^ complex ions in a single pass on the filter such that citrate accumulation (C_Max_ > 2.5 mM) will not occur in any patient with > 6 L/h liver citrate clearance, which encompasses about 90% of critically ill patients in our ICUs that serve a large liver transplant program.
5. A method to detect significantly compromised liver metabolism (ie citrate clearance < 6 L/h) that could potentially affect systemic iCa within the first 6 h of CKRT initiation. A decrease of iCa greater than 0.3 mmol/L at hours 2, 4 or 6 prompts a switch to the “shock” protocol” (> 75% single pass citrate extraction) and before the development of clinically dangerous low systemic iCa levels.
6. Equal QD and QRF settings for equal scale use on our local CKRT device the Prismaflex which has 2, separate 5-Kg scales for dialysate- and replacement fluids for ICU nurse convenience and for single pass citrate removal of about 40%. This strategy is feasible without excessive post-filter hemoconcentration if the systemic Hb level is < 12 g/dL. With Hb levels above this value the combined QD + QRF flow rate can be split in a 2:1 (Hb < 14 g/dL) or 3:1 (Hb > 14 g/dL) QD: QRF ratio to avoid TMP spikes due to hemoconcentration with post-dilution filtration.
7. Changing the effluent dose during the course of CVVHDF-RCA is simple: the prescriber notes the current (old) total effluent flow rate and QCa, then selects a new row of settings from Table 1 corresponding to the desired new total effluent flow, and finally sets the new QCa = old QCa x (new effluent flow/old effluent flow). Using this method, we typically see < 0.1 mM fluctuations in the systemic iCa after adjusting the effluent dose.
8. The protocol allows two goal systemic ionized calcium ranges 1.05–1.25 or 1.2–1.4 mM with their dedicated calcium titration table one of which is chosen at the discretion of the critical care team. The higher calcium dosing table is adopted when ionized calcium levels greater than 1.2 mmol/L are desired by the critical care team in patients with refractory shock [14]. This approach will minimize the administration of calcium bolus infusions which only transiently elevate systemic ionized calcium levels during CKRT-RCA and may confound dosing of the continuous calcium infusion.

Multiple CKRT-RCA protocols were developed for different CKRT modalities using different citrate solutions, dialysate/replacement fluids and a wide range of operational parameters [6–8, 15–27], significantly affecting acid-base balance and electrolytes levels [18, 22, 26, 27]. Complications related to citrate or citrate formulations include hypercalcemia, hypocalcemia, hypernatremia, metabolic alkalosis or acidosis, particularly in patients with shock and severe liver dysfunction [28, 29].

Hypocalcemia is the most feared electrolyte complication of RCA as it can precipitate life-threatening arrhythmias [30–32], decreased myocardial contractility [33], and hypotension [34]. Incident cases of ionized hypocalcemia were rare in our cohort for three reasons. First, the personalized initial calcium dosing ensures a neutral CKRT Ca mass-balance. Second, only patients with normal or moderately impaired liver metabolism (i.e. liver citrate clearance > 6 L/hour) were treated with this protocol based on initial triaging criteria and therefore systemic citrate accumulation to > 2.5 mM was unlikely in these patients. The maximum recorded tCa/iCa ratio in any 24-h period was 2.55 suggesting systemic citrate accumulation was maintained within a clinically acceptable level in all patients. Finally, in those with frequent albumin infusions, the systemic iCa checks every 6 h allow for adjustments to the QCa without any development of clinically concerning ionized hypocalcemia. Incident cases of hypercalcemia were largely avoided due to personalized initial calcium dosing and every 6 h systemic iCa checks.

Hypernatremia is a potential complication related to the use of hypertonic citrate solutions that could in part be avoided by using low-sodium concentration dialysate and/or replacement fluids [16, 18, 19, 28, 35]. However, beyond the sodium content of CKRT and the hypertonic citrate solutions, other CKRT operational parameters may impact the steady state sodium level such the citrate solution flow rate, the effluent rate and the blood flow rate. We minimized this risk by using ACDA which is relatively less hypertonic (Na 225 mM/citrate 113 mM) than 4% sodium citrate (Na 408 mM/citrate 136 mM) and by using a commercial CKRT fluid with 136 Na level which we pre-calculated as optimal for the fixed flow settings in Table 1. Mild increases in serum sodium were expected as rare spiking of the CKRT fluid from 25 up to 35 HCO3 resulted in final CKRT fluid Na levels up to 146. Acid-base control was adequate.

Hypophosphatemia can complicate CKRT with or without RCA when phosphate is not adequately supplemented. Its’ occurrence in patients undergoing CKRT has been associated with adverse clinical outcomes including prolonged mechanical ventilation requirements, longer hospital length of stay, and mortality [36–38]. Phosphate is supplemented either as a standalone oral or parenteral treatment or as an additive to CKRT solutions [39]. In our program, hypophosphatemia was abrogated by spiking commercial CKRT fluids to a phosphate concentration of either 0.68 or 1.36 mmol/L.

Hemofiltration circuits ideally are changed every 72 h since tubing integrity and solute clearance are not guaranteed after this time interval. They frequently last far less time because of filter clotting resulting in blood loss, lost time for solute control, increased nurse workload, and increased cost. Several observational studies [15, 40, 41] and clinical trials have shown that filter lifespan with RCA was significantly higher compared to heparin [6, 7, 17, 42, 43]. Reported filter lifetimes with RCA varied widely in these studies likely related to differences in protocol design. In our program, the CKRT circuits are allowed to run for up to 96 h. The median filter life exceeded 72 h in this study. The high hemofilter lives are likely afforded by the higher than traditional (around 1:40) ACDA-to-blood flow ratios (both in ml/min) and the use of Ca-free CKRT fluids. The ACDA-to-blood flow (both in ml/min) ratios used in this protocol are 1:24 and 1:30 at blood flows of 100 and 150 ml/min, respectively. These ratios ensure that the circuit iCa is < 0.4 mmol/L even in patients with low hematocrit when systemic iCa is < 1.4 mM and albumin is < 5 g/dL based on studies of human plasma and citrate interactions [44, 45].

There are limitations to the “Non-shock protocol” and to this study. First, the protocol is not advised to be adopted in all ICU patients. It should be avoided in patients with presumed absent liver metabolism (e.g. systemic lactate ≥10 mM in patients with shock, or requirement of fresh frozen plasma (FFP) drip to keep the INR < 3 in patients with cirrhosis, or a diagnosis of acute liver failure/shock liver. In our center, those patients are triaged to receive the “Shock protocol” which is designed to maximize single pass citrate extraction on the filter [10]. Second, our center has several intensive care units and therefore oversees large volumes of CKRT treatments. Institutional resources are expended to maintain ICU nurse CKRT skill sets at high proficiency. It is possible that nurse expertise may have contributed to our favorable filter outcomes. Third, although the filter life exceeded 72 h, complete prevention of clotting was not possible. We attribute this to intermittent stoppage of the citrate infusion delivered by the Pre-Blood-Pump of the Prismaflex machine with effluent-, ACDA-, dialysate- and substitution fluid bag changes while the machine keeps the blood pump running, resulting in temporarily normalized > 1 mM circuit iCa levels and the possibility of clotting. Finally, all the treatments were delivered using HF1400 hemofilter sets in the “Non-shock” protocol. The filter is a high flux filter with a surface area of 1.4 m^2^. It is expected that small solute fluxes are likely to be similar on other high flux filters with a similar or greater surface area. During the Covid-19 pandemic in the setting of shortages of HF1400 filter sets, we did utilize other filter sets such as the Oxiris membrane (1.5 m^2^) and the smaller surface area HF1000 (1.0 m^2^) and M100 filter (0.9 m^2^). No differences in solute fluxes were noted even with the smaller surface area filters. The latter is likely because at least half of the small solute clearance is provided through convective clearance which is less affected by surface area reduction and because at the low, up to 2.5 L/hour dialysate flows used even a smaller membrane surface area does not become limiting of diffusive small solute clearance.

## Conclusion

The development of protocols based on solute kinetic analysis and the introduction of integrated calcium- and citrate infusion systems and RCA-dedicated software could help simplify RCA delivery and support broader and safer use of RCA without contraindications. Our unique approach for ICU providers with CVVHDF-RCA fluid compositions and settings pre-calculated for optimal solute kinetic outcomes is effective in maintaining circuit patency as a high ACDA to blood flow rate is used in combination with Ca-free CKRT fluids. The prescribing algorithm can be easily and mechanistically followed by ICU providers without the need for broad experience in solute kinetic analysis, and this contributes to the safer and simpler use of CVVHDF-RCA for the vast majority of ICU patients with liver citrate clearance at least 6 L/h.

## Data Availability

All data generated or analyzed during this study are included in this published article
